# Evidence of a two-dimensional glass transition in graphene: Insights from molecular simulations

**DOI:** 10.1038/s41598-019-41231-z

**Published:** 2019-03-14

**Authors:** R. Ravinder, Rajesh Kumar, Manish Agarwal, N. M. Anoop Krishnan

**Affiliations:** 1 Department of Civil Engineering, Indian Institute of Technology Delhi, Hauz Khas, New Delhi 110016 India; 2 Computer Services Center, Indian Institute of Technology Delhi, Hauz Khas, New Delhi 110016 India; 3 Department of Materials Science and Engineering, Indian Institute of Technology Delhi, Hauz Khas, New Delhi 110016 India

## Abstract

Liquids exhibit a sudden increase in viscosity when cooled fast enough, avoiding thermodynamically predicted route of crystallization. This phenomenon, known as glass transition, leads to the formation of non-periodic structures known as glasses. Extensive studies have been conducted on model materials to understand glass transition in two dimensions. However, despite the synthesis of disordered/amorphous single-atom thick structures of carbon, little attention has been given to glass transition in realistic two-dimensional materials such as graphene. Herein, using molecular dynamics simulation, we demonstrate the existence of glass transition in graphene leading to a realistic two-dimensional glassy structure, namely glassy graphene. We show that the resulting glassy structure exhibits excellent agreement with experimentally realized disordered graphene. Interestingly, this glassy graphene exhibits a wrinkled but stable structure, with reduced thermal vibration in comparison to its crystalline counterpart. We suggest that the topological disorder induced by glass transition governs the unique properties of this structure.

## Introduction

Discovery of graphene^1^, a two-dimensional honeycomb structure of carbon atoms, marked the beginning of a new era in nanotechnology. Due to its superior electronic, chemical, physical, and mechanical properties, graphene holds promise in a wide range of applications^2^. The success of graphene motivated research in other two-dimensional crystalline materials such as h-BN^3^, MoS_2_, WSe_2_^4^, and their nanotubes. Despite the significant progress in two-dimensional crystalline materials, its disordered counterparts remain poorly explored. Studies on the two-dimensional glassy equivalents for graphene and isoelectronic materials are relevant from both fundamental and practical standpoints because of the following reasons. (i) Fundamental mechanisms governing the glass transition in two-dimensional systems, and its difference from three-dimensional systems, are still poorly understood^5^. Graphene presents a unique realistic two-dimensional material to investigate the nature of glasses and glass transition in two dimension. (ii) Changing the perfect honeycomb structure in graphene by inducing defects affects its electronic^6–8^, and mechanical properties^9–14^ and increase its chemical reactivity^15,16^. Since glasses are considered as archetypical disordered materials, two-dimensional glasses may possess unique properties in comparison to their crystalline counterparts. Therefore, a fundamental understanding on realistic two-dimensional glasses can potentially lead to nano-engineered layered materials with tunable properties.

Although the terms *glassy*, *disordered*, *amorphous*, and *non-crystalline* are interchangeably used, glasses represent a unique non-equilibrium state distinct from other disordered states. To explicate this, a meaningful definition of glass was provided by Varshneya and Mauro^17^ as *“solids having a non-crystalline structure*, *which continuously convert to a liquid upon heating”*. Further, an improved and precise definition for glass was proposed recently by Zanotto and Mauro^18^ as *“a nonequilibrium*, *non-crystalline condensed state of matter that exhibits a glass transition*. *The structure of glasses is similar to that of their parent supercooled liquids (SCL)*, *and they spontaneously relax toward the SCL state*.*”* Note that glass transition is marked with a continuous evolution of thermodynamic quantities such as density, molar volume, and enthalpy as a function of temperature with the absence of any discontinuities^19^. This behavior makes it distinct from any first-order phase transitions such as melting or evaporation. In the present work, we rely on this definition of glass. Most of the studies on two-dimensional glasses and glass transitions are restricted to model materials such as LJ particles, or simple systems constrained in two-dimensions such as colloids or granular materials^5,20–31^. While they are extremely valuable to understand the nature of glass transition in two dimensions, the transferability of these model systems to realistic two-dimensional materials such as graphene is not clear.

Recent studies have successfully demonstrated the experimental realization of a Zachariasen carbon monolayer employing low-pressure chemical vapor deposition (LPCVD)^32^. Further, disordered graphene has been achieved using alternate methods such as CVD^33^, ion irradiation^16,34,35^ and electron irradiation^36,37^. The disordered graphene thus obtained have been shown to exhibit notably distinct properties as compared to the crystalline graphene (CG)^37^. For example, the Zachariasen carbon monolayer exhibits a unique Anderson-insulating behavior in contrast to the crystal^37^. These experimentally realized structures have been simulated using different computational techniques such as geometrical modelling and bond-switching^38–40^. Despite the realization of these disordered structures including Zachariasen monolayer, none of these studies demonstrate the existence of glass transition in graphene. As such, a fundamental understanding on the nature of these disordered states, and the consequence of such disorder on the structure and properties of graphene remain unexplored.

Here, using molecular dynamics simulation, we demonstrate the existence of glass transition in graphene leading to a two-dimensional glassy structure, namely glassy graphene (GG). We show that this glassy structure exhibits close similarity to experimentally realized disordered graphene. Interestingly, GG exhibits a unique wrinkled but stable structure different from that of CG. Further, the structure exhibits reduced out-of-plane fluctuations in comparison to the crystal. We suggest that the disordered structure formed upon glass transition induces the wrinkled structure with reduced ripples.

## Results

### Glass transition

**Figure 1:**
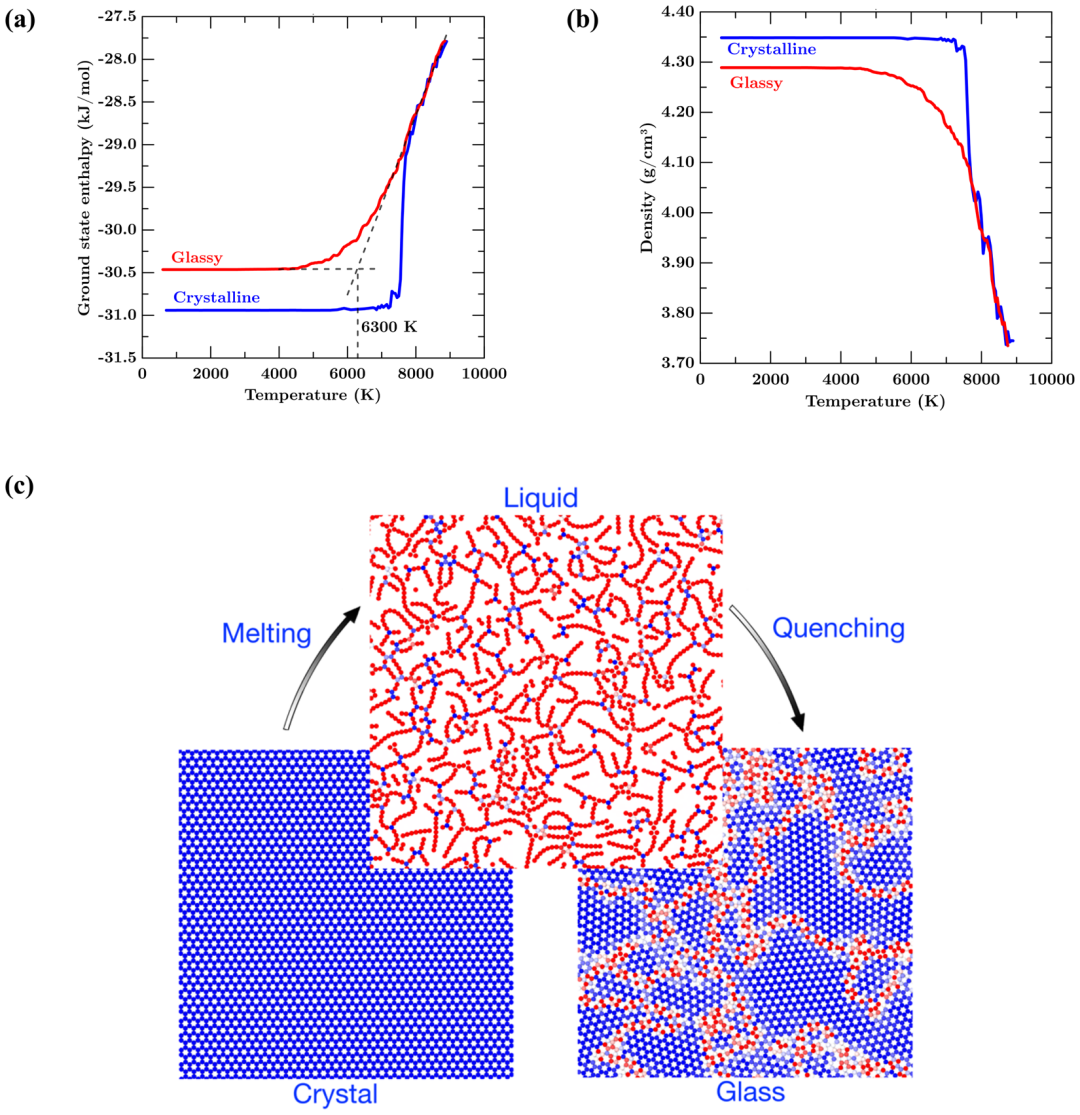
(**a**)Ground state enthalpy (kJ/mol), and (**b**) Density (g/cm^3^) with respect to temperature (K) for crystalline and glassy graphene. (**c**) Visual representation of the structure of crystal, liquid, and glass graphene.

To establish glass transition in graphene, we analyze the evolution of ground-state enthalpy and density. Figure 1(a,b) show the variation in ground-state enthalpy and density, respectively, of graphene as a function of temperature. Upon heating the CG, the ground-state enthalpy remains constant up to 7500 K confirming that the system retains the crystalline phase up to this temperature. This is because the crystal, when removed of all the thermal fluctuations, will have a unique minimum energy corresponding to the perfect hexagonal lattice structure. This behavior is further confirmed from the density of the system, which exhibits a constant value in this temperature range (see Fig. 1(b)). Note that the density is obtained by considering the thickness of graphene sheet as 3.4 Å, the interlayer separation distance^41^. At 7500 K, we observe a sudden increase in the *H*_0_ suggestive of a first-order melting transition. The abscissa corresponding to the discontinuity in *H*_0_ corresponds to the enthalpy of fusion upon melting. The phase transition is further confirmed by the discontinuity in density at the same temperature leading to a lower value. This is due to the change in the structure from crystalline to that of a disordered liquid-like structure. A visual representation of the structures obtained from the trajectory of the atoms during simulation is shown in Fig. 1(c). After melting, the *H*_0_ of graphene increase monotonically with the temperature representative of the equilibrium liquid melt. The heating is continued up to a temperature of 9000 K to obtain the equilibrium thermodynamic quantities, density and *H*_0_, with respect to temperature. It should be noted that 7500 K, representing the melting temperature *T*_*m*_, of the graphene sheet is over-predicted^42,43^. This is attributed to the two-dimensional nature of the simulation wherein the out-of-plane fluctuations are suppressed. Further, the periodic boundary conditions essentially represent an infinite graphene sheet devoid of any defects, delaying the initiation of the melting. These effects compounded, lead to an increased *T*_*m*_ for graphene in comparison to previous studies^42,43^.

At 9000 K, we start cooling the liquid graphene at a rate of 100 K/ps (see Methodology section). Upon cooling, we observe that the density and *H*_0_ decreases monotonically with the temperature (see Fig. 1(a,b)). Interestingly, in contrast to the behavior upon heating, we observe that the density and *H*_0_ doesn’t exhibit any discontinuity. Instead, it decreases continuously and gradually before plateauing to a constant value at lower temperatures of about 5500 K demonstrating glass transition. Indeed, while cooling, we observe a change in slope of the density and *H*_0_ at 6300 K (see Fig. 1(a)), where the system falls out of equilibrium. This temperature, obtained by interpolating the liquid and glassy states, represent the fictive temperature of the system and the onset of the glassy regime, before which the system is in the state of an equilibrium supercooled liquid^19^. Beyond this regime, the system exhibits a glassy state with the density and *H*_0_ remaining fairly constant at low temperatures. In addition, as expected, we observe that final structure of GG at 300 K has a higher ground state enthalpy and a lower value of density as compared to CG (see Fig. 1). Visualization of the complete atomic trajectory corresponding to the melting and glass transition of graphene is presented in the Supplementary Material (see video S1). Indeed, the disordered nature of the glassy structure leads to a less dense material having a higher value of *H*_0_. Note that this behavior, in agreement with the postulates of Zachariasen^44^, has been observed in other archetypical glass forming systems such as silica^45^. Overall, by analyzing the thermodynamic response of graphene over a wide range of temperatures, we conclusively demonstrate the existence of glass transition in graphene.

### Structure of glassy graphene

**Figure 2:**
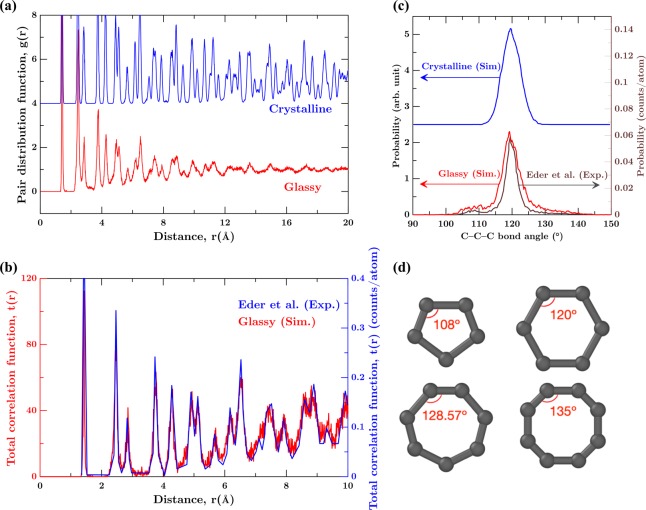
(**a**) Pair distribution function g(r) with respect to distance (Å) for GG and CG. (**b**) Total correlation function t(r) with respect to distance (Å) for GG compared with irradiated graphene structure by Eder *et al*.^37^ (**c**) Bond angle distribution for CG, GG. Angular distribution of C–C–C angles for irradiated graphene obtained by Eder *et al*.^37^ is plotted for comparison. (**d**) Interior angles for 5-, 6-, 7-, and 8-membered rings occurring in GG.

Now, we focus on the structure of the GG obtained following the glass transition. First, we analyze the short-range order (<3 Å) of the structures obtained at 300 K. Figure 2(a) shows the pair distribution functions (PDFs) of GG and CG. The position of the first peak in the PDFs of CG and GG coincide, suggesting that the average C–C bond length in the first coordination shell is similar in both the structures. However, there is a significant broadening in the second and third peaks of the PDF corresponding to GG. This broadening indicates an increased disorder in the structure of GG in comparison to CG. Further, we observe that the PDF of GG eventually converges to a value of 1, confirming a disordered glassy structure in the long range (>12 Å).

In order to ensure the realistic nature of the structure obtained, we compare the PDF of GG with that of irradiated graphene obtained experimentally^37^. In experiments, Note that while irradiated and vitrified structures exhibit some differences, glasses have been used as surrogates to estimate the extent of disordering induced up on irradiation^45,46^. Further, it has been shown in the case of silica that hyperquenched liquid exhibit a close match to irradiated structures in the short-, medium-, and long-range order^45^. Figure 2(b) shows the total correlation function (TCF) of GG with respect to the irradiated graphene obtained experimentally^37^, for a density. First, we observe that the position of the peaks in TCFs obtained from the simulations exhibit an excellent match with the experimental structure. Further, the relative magnitudes of the peaks of the TCFs also exhibit a close match suggesting the similarity of GG with the structure of irradiated graphene. In other words, the short-range order in irradiated graphene and GG exhibits a close match suggesting similar degree of disorder.

It should be noted that the glassy structure obtained here is different from the canonical three-dimensional glasses such as glassy silica^19,47,48^. Rather, we observe that the glassy structure here is an arrangement of irregular nanocrystalline domains, with significant defects accumulated at the domain boundaries. Owing to the non-equilibrium nature of glasses, the nanocrystalline structure obtained herein, could be strongly dependent on the thermal history^47,49,50^. To analyse the effect of thermal history, we quenched the graphene at a higher cooling rate of 1000 K/ps. We observe that the structure obtained by faster cooling exhibit higher enthalpy, lower density, and consequently, higher disorder (see Supplementary Material). This is consistent with earlier observations in silicate glasses which shows that a higher cooling rate results in a glass with increased fictive temperature and higher degree of disorder^47,49^. Further, we compare the structure obtained from higher cooling rate with the experimental results of irradiated graphene for a higher dosage^37^. We observe an excellent match with the experimental structure for the GG with increased disorder as well (see Supplementary Material). Overall, these results suggest that while the structure of graphene can be significantly affected by the thermal history, the structures exhibit a close match with the corresponding experimentally realized structures.

To assess the angular order, we plot the C–C–C bond angle distribution of CG and GG (see Fig. 2(c)). For CG, the bond angle distribution exhibits a unimodal distribution with a peak value at 120°, the preferred angle for the hexagonal carbon ring in CG. In the case of GG, there is a significant broadening of the peak in the bond angle distribution (see Fig. 2(c)), which indicates deviations from the perfect hexagonal rings. In particular, we note small shoulders at 108° and 128°. These angles correspond to the presence of 5- and 7-membered rings in GG, respectively (see Fig. 2(d)). Further, the bond angle distribution of GG is compared with the experimental results for irradiated graphene^37^. Similar, to the total correlation function, we observe a close match in the bond angle distribution of GG and irradiated graphene^37^. In particular, the shoulders at 108° and 128°, representative of the 5- and 7-membered rings exhibit an excellent agreement in the irradiated graphene^37^ and GG. This indicates that the irradiated graphene and GG exhibit similar translational and orientational short range order. Altogether, the results suggest that the GG obtained after the glass transition indeed correspond to a realistic non-crystalline structure.

**Figure 3:**
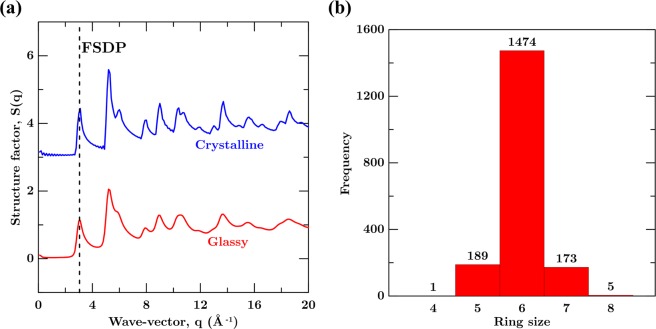
(**a**) Structure factor S(q) with respect to wave-vector q (Å^−1^) for GG and CG. (**b**) Distribution of rings for GG.

Now, we focus on the medium range order (3 Å to 12 Å) in GG. To this end, we analyze the structure factor and the distribution of carbon ring size in GG. Figure 3(a) shows the structure factor, S(q), for CG and GG. We observe that the S(q) for GG exhibit wider peaks in comparison to the crystalline counterpart. In particular, the full width at half maximum (FWHM) of the first sharp diffraction peak (FSDP), which captures structural correlation in medium-range order, is broader in GG suggesting an increased disorder at medium range^51,52^. In order to quantify this, we compute the FWHM as 0.34 Å^−1^ and 0.53 Å^−1^ for crystalline and glassy structures, respectively. The increased FWHM of the GG suggests that the structure is significantly disordered in the medium range^53^. To confirm this further, we compute the ring distribution in GG as shown in Fig. 3(b). As expected from the C–C–C bond angle distribution, we find a significant number of 5- and 7-membered rings GG. Note that while smaller rings below 5-membered could induce significant internal stress, larger rings above 7-membered could destabilize the sheet-like structure. This behavior has been observed in other systems such as silica and sodium silicate glasses as well^45,54,55^. As such, the optimum ring size around 6 is preferred in GG. Overall, this confirms that the medium range order in GG is notably distinct from CG as well.

### Stability of glassy graphene

**Figure 4:**
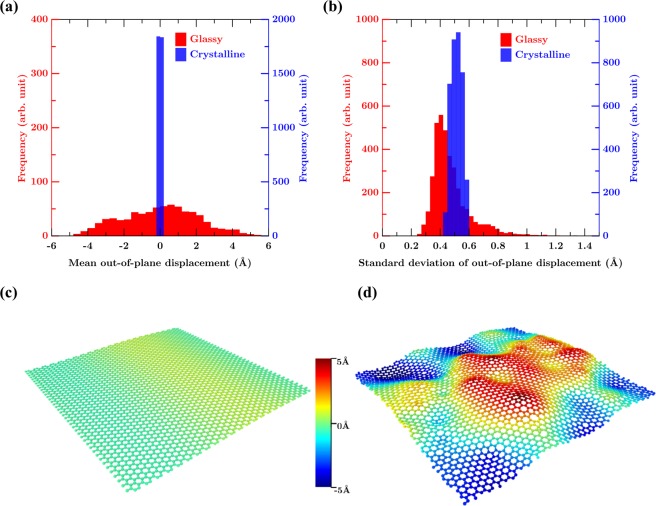
Distribution of (**a**) mean, and (**b**) standard deviation of out-of-plane displacement of C atoms in GG and CG at 300 K. (**c**) Temporal mean of out-of-plane atomic positions in CG and (**d**) GG structure at 300 K. Note that the GG exhibits a static wrinkled structure in contrast to its crystalline sheet. Coloring scheme is based on the out-of-plane displacement.

Finally, to ensure the realistic nature of the structure obtained by glass transition, we investigate the stability of GG in 3D at 300 K. To this extent, we equilibrate the GG at 300 K for 1 ns, allowing for displacements in the third dimension. Figure 4(a) show the mean out-of-plane displacement in CG and GG. We find that the GG structure is stable, although exhibiting some intrinsic ripples with respect to time as in the case of CG. Interestingly, we note that, in addition to the thermally driven intrinsic ripples, the 3D relaxed structure of GG exhibits a static wrinkled structure. This is evident from the mean out-of-plane displacement, wherein GG exhibits a multimodal distribution suggestive of a static wrinkled structure (see Fig. 4(d)). This is in contrast to the CG which exhibits a sharp unimodal distribution (Fig. 4(c)). We note that the wrinkled structure is primarily due to the local defects present in the structure. The additional energy caused by the formation of defects is released by inducing a local curvature in the geometry. Similar behavior has been observed in carbon nanotubes and graphene with Stone-Wales defect as well wherein the defect induces a local curvature in the structure^9,11,56^.

In order to analyze the dynamic ripples, the standard deviation of the out-of-plane displacements are plotted in Fig. 4(b). In contrast to the mean, we observe that the standard deviation of GG is notably lower than that of CG. This suggests that the out-of-plane fluctuations in GG is significantly suppressed in comparison to its crystalline counterpart. This reduction is attributed to the wrinkled structure wherein the local curvature formed by the local topological defects suppress the long wave-length fluctuations in GG. Such behavior has been observed experimentally as well^14^, which could result in an enhancement of elastic properties and strength of the GG^11^. Overall, these results suggest that the intrinsic ripples in GG can be controlled by tuning the topological order in the wrinkled structure.

## Discussion

Our study demonstrates the existence of glass transition in graphene which leads to a two-dimensional glassy structure. Specifically, we show that there is continuous evolution of density and enthalpy which confirms the glass transition, and that the glassy structure is stable and non-crystalline in nature. Reconciling our simulation results with previous experimental studies, we confirm that GG exhibits a realistic structure with topological disorder. We also show that GG exhibits a wrinkled structure with reduced thermal vibrations. This observation is in agreement with previous experiments which show that topological defects suppress long wavelength fluctuations in graphene^11,14^ reducing the overall fluctuation. Overall, our results demonstrate that graphene can exhibit glass transition, which can be experimentally realized using controlled ion-irradiation and LPCVD.

Owing to the non-equilibrium nature of glasses, the properties of the final structure are strongly dependent on the thermodynamic history including cooling rate, and applied pressure^49,57^. To this extent, LPCVD offers a unique methodology to tune the structure of GG by controlling the rate of deposition and temperature. Similarly, controlled electron-irradiation could also be used to generate defective structures through an atom-by-atom transformation process^37^. As such, these methods could be used to develop topologically engineered GG with variable degrees of disorder. Indeed, it has been shown that defects can enhance the mechanical properties of graphene^11,14^. It would be worthwhile to investigate how glass transition can potentially alter the elastic properties and strength of graphene. This is particularly relevant in applications where the out-of-plane fluctuations should be controlled, for example, strain sensors, or graphene-based NEMS systems.

It is worthwhile to mention a few fundamental open questions that this study raises. Although it has been observed that graphene undergoes glass transition, the driving force inducing this mechanism for a homonuclear system is not understood. Owing to its simplicity, graphene presents an excellent system to study the mechanisms governing glass transition versus crystallization in two dimensions. Further, the present study can be extended to other two-dimensional materials such as h-BN or MoS_2_. This will be crucial in understanding the role of frustration^58^ in heteronuclear systems versus homonuclear systems towards glass transition. It has been suggested that the rapid slowing down of the system at a particular temperature, known as the fictive temperature, could be associated with dynamical heterogeneities or static correlation lengths^29,59,60^. Investigating the kinetics and dynamics of graphene and h-BN systems will shed light on these issues, leading to a better understanding of the structural signatures associated with glass transition.

## Methodology

### Glass preparation

We prepare GG following the conventional melt-quench method^19,46,49,61^ in molecular dynamics (MD) simulations using the open-source package LAMMPS^62^. Simulation of glass transition requires a sophisticated interatomic potential, which can accurately describe both pristine and disordered configurations. Further, it should be able to realistically model the formation over- or under-coordinated species with defective bond angles that are likely to form during glass transition. To this end, we employ the AIREBO^63^ potential, which has been extensively used to investigate pristine and defective graphene and carbon nanotubes^9,64,65^. Note that AIREBO is a bond-order based potential which dynamically compute the potential energy of the system based on the local environment of the atoms^63^. The glassy structure is then simulated as follows.

First, the hexagonal crystalline structure of graphene sheet with a lattice parameter of 2.46 Å is generated. Periodic boundary conditions are applied in the in-plane directions, while non-periodic boundary conditions are applied in the direction normal to the sheet. This is to avoid potential self-interactions of the graphene sheet along the normal direction. Note that, initially, the simulation is restrained to two dimensions, preventing any out of plane fluctuations for the sheet. Now, the system is gradually heated in the *NPT* ensemble with a rate of 100 K/ps to a temperature of 9000 K, wherein it forms a two-dimensional liquid melt. A nominal pressure of 3 GPa is applied to prevent the system from “explosion”, wherein the volume of the system could increase indefinitely with time. The system is equilibrated at this temperature for 1 ns. After ensuring equilibrium, the system is gradually cooled from 9000 K to 300 K with a cooling rate of 100 K/ps in the *NPT* ensemble. The final structure is relaxed at 300 K for 1 ns to ensure equilibration of the two-dimensional glass obtained. Finally, the stability of the sheet in 3D is ensured by removing the restraint in the third dimension. The two-dimensional glass obtained is equilibrated in 3D at 300 K for 1 ns, allowing out of plane fluctuations to obtain a realistic glassy graphene structure.

### Ground-state enthalpy computation

In order to demonstrate the phase/glass transition occurring upon heating or cooling, respectively, we investigate the evolution of the enthalpy of the system. At finite temperature, random thermal vibrations contribute to the instantaneous potential energy of the system inducing uncertainty in the measurements of enthalpy. To mitigate this issue, we compute the ground-state enthalpy *H*_0_, which removes thermal fluctuation and calculates the enthalpy due to inherent structure. Thus, *H*_0_ correspond to enthalpy of system at 0 K. This is achieved by the process of energy minimization^62,66^ at zero pressure. This method removes the contribution of thermal energy from the calculated enthalpy by ensuring that all the atoms reach their local minimum. Note that energy minimization leads to local, and not global, minimum of the enthalpy of the system. Thus, this method can be used to obtain the *H*_0_ at any finite temperature. To calculate the ground-state enthalpy, system is instantaneously cooled to a temperature close to 0 K and relaxed in the *NPT* ensemble for 1 ns. This is followed by energy minimization using the conjugate gradient scheme at 0 K. This process is repeated at every 50 K to obtain the systematic variation of ground state enthalpy with respect to temperature.

### Pair-distribution functions

The transitional symmetry in a system is reflected by the pair distribution function (PDF), which describes the probability of finding an atom at a given distance from the reference atom. Since CG and GG both systems are 2D, we calculate the PDF for using

$$g(r)=\frac{\mathop{\mathrm{lim}}\limits_{{\Delta }r\to {\rm{0}}}\frac{{N}_{r}}{\pi {({r}+{\Delta }r)}^{2}-{\pi }{{r}}^{2}}}{\rho }$$

where, *g(r)* is the pair distribution function, *r* is the radial distance from reference atom and *ρ* is the average atom density and *N*_*r*_ is number of atoms between *r* and *r* + *Δr*.

### Orientational order

The orientational symmetry of the system is computed using the bond orientation order parameter. In particular, we focus on the triatic order of the system, since one carbon atom is connected to 3 carbon atoms in the crystalline graphene sheet. The triatic order *q*_3_, is given by

$${q}_{3}=\frac{1}{n}{\sum }^{}{e}^{{\rm{3}}i{\theta }{{r}}_{{ij}}}$$

where, *n* is the number of nearest neighbors of the central atom. Note that the value of *q*_3_ varies between 0 and 1, where 1 corresponds to an atom bonded symmetrically with its neighbors having an internal angle of 120°.

### Ring computations

Ring computations were done using the opens-source package R.I.N.G.S^67^. Here, only the primitive rings (or irreducible rings) which cannot be decomposed into smaller rings are enumerated.

## Supplementary information

Supplementary Material

S1
