# Supplementary material for: Evidence of a two-dimensional glass transition in graphene: Insights from molecular simulations
Source: Sci Rep. 2019 Mar 14;9:4517. doi: 10.1038/s41598-019-41231-z (PMC6418284; doi:10.1038/s41598-019-41231-z)
Supplement: Supplementary file 1 — Supplementary Material [file 41598_2019_41231_MOESM1_ESM.docx]

**Evidence of a two-dimensional glass transition in graphene: Insights from molecular simulations**

*R. Ravinder^1^, Rajesh Kumar^1^, Manish Agarwal^2^, N. M. Anoop Krishnan^1,3*^*

^1^Department of Civil Engineering, Indian Institute of Technology Delhi, Hauz Khas, New Delhi 110016, India

^2^Computer Services Center, Indian Institute of Technology Delhi, Hauz Khas, New Delhi 110016, India

^3^Department of Materials Science and Engineering, Indian Institute of Technology Delhi, Hauz Khas, New Delhi 110016, India

^*^Corresponding author: N. M. A. Krishnan ([krishnan@iitd.ac.in](mailto:krishnan@iitd.ac.in))

**Supplementary Material**

**Effect of cooling rate**

In order to study the effect of cooling rate, we perform melt-quenching simulation (see Methodology) for two different cooling rates, namely, 100 K/ps and 1000 K/ps. We observe that a higher cooling rate (i.e. 1000 K/ps) lead to a more disordered graphene sheet compared to lower cooling rate (100 K/ps). Further, the final configuration corresponding to cooling rate of 1000 K/ps have lower density and higher ground state enthalpy in comparison to the configuration obtained with a cooling rate of 100 K/ps.

| 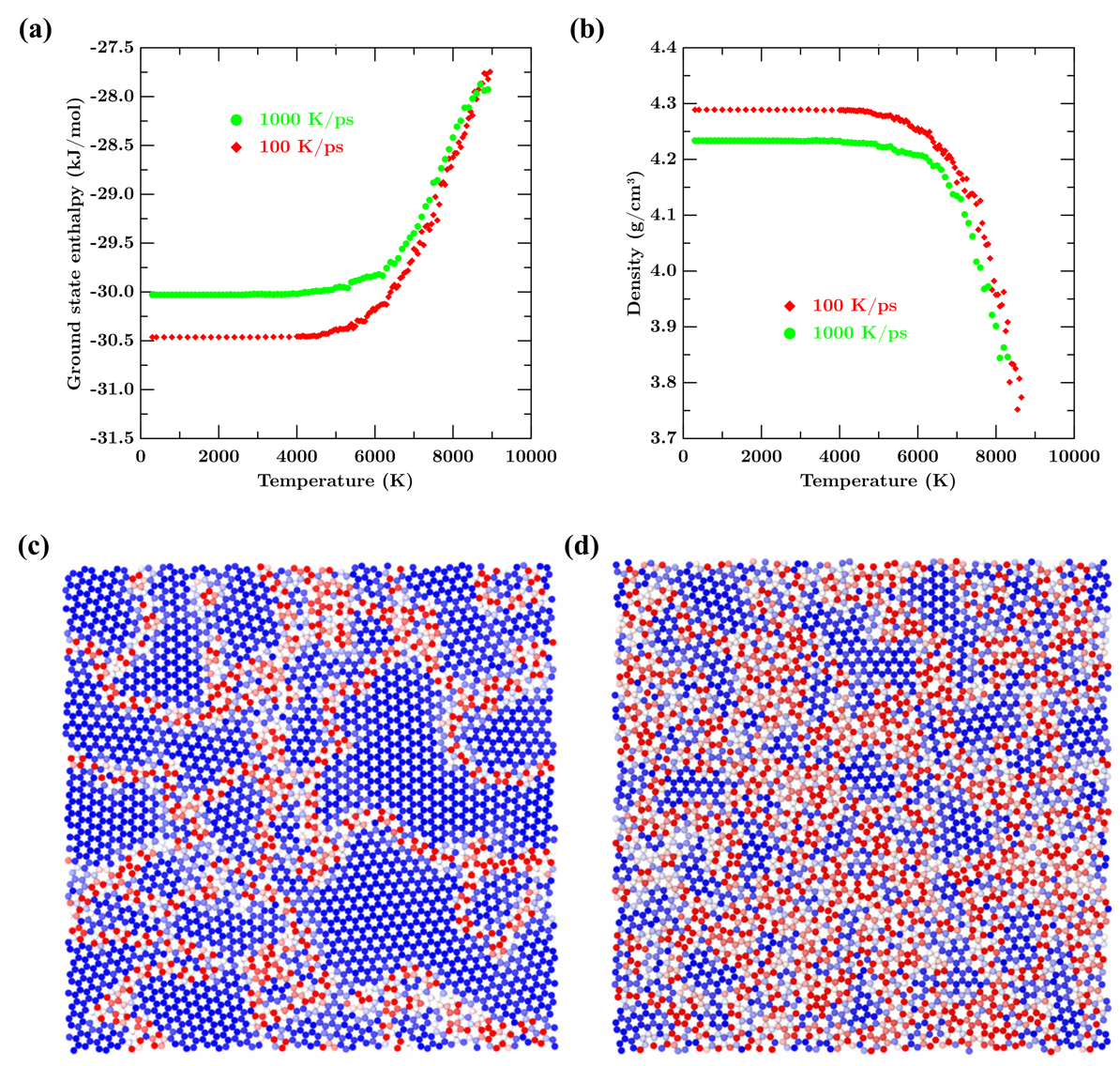 |
| --- |
| **Figure S1. (a)** Ground state enthalpy (kJ/mol), and **(b)** Density (g/cm^3^) with respect to temperature (K) cooling rate of 100 K/ps and 1000 K/ps. Atomic configuration of the structure of graphene sheet at 300 K for cooling rate of **c)** 100 K/ps and **d)** 1000 K/ps. |

**Structure of glassy graphene**

We analyze pair distribution function (PDF) for both the final configuration (for cooling rate of 100 K/ps and 1000 K/ps). As shown in Figure S2, the first peak for 100 K/ps and 1000 K/ps occurs at the same distance, indicating the average bond length in both the configurations are same. Further, we observe that the second and third peaks are broader in case of cooling rate 1000 K/ps, suggesting an increased degree of disorder for higher cooling rate. Eventually, both the PDFs assumes a value of 1 at large distances, representing a disordered structure with no long range order.

| 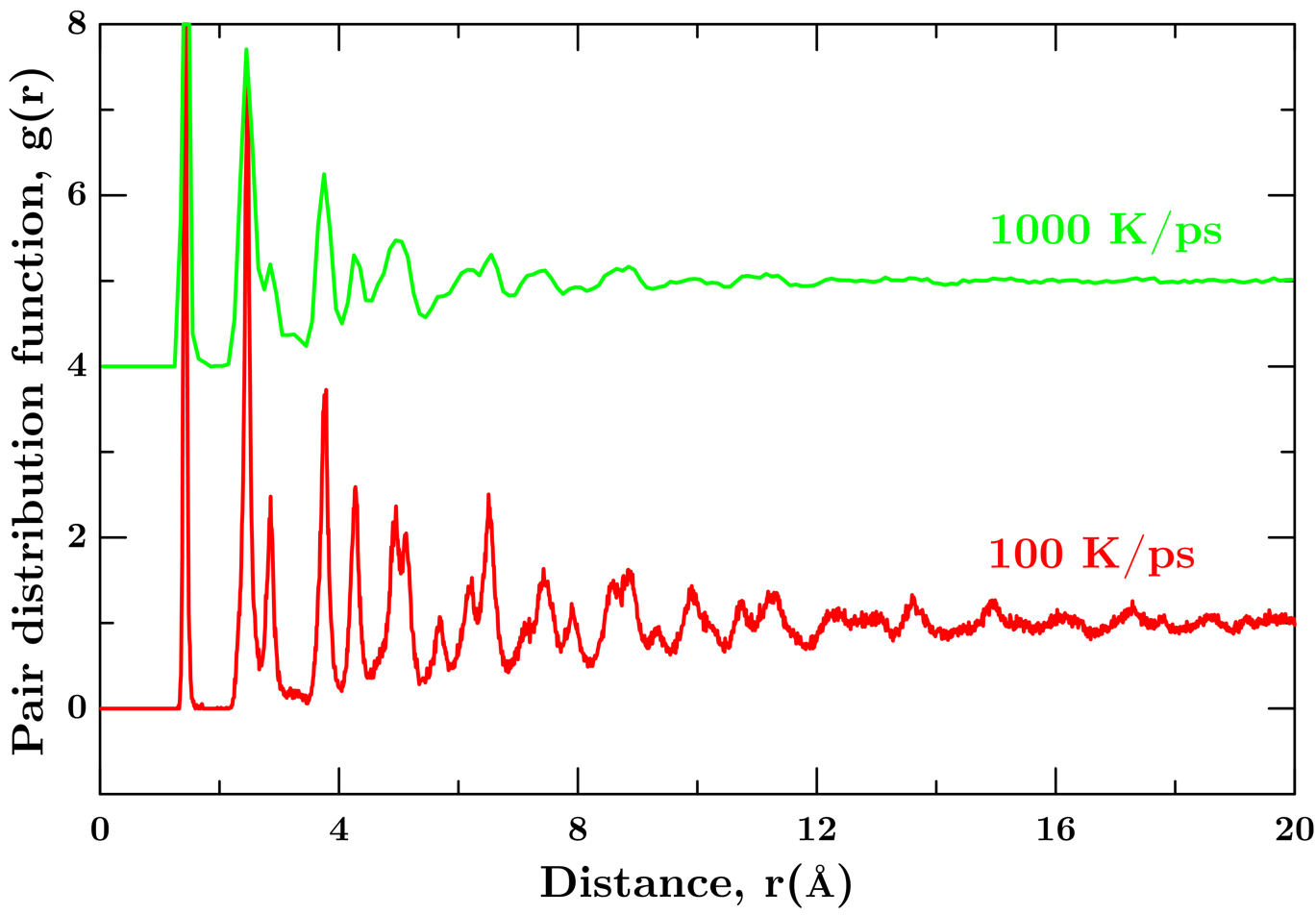 |
| --- |
| **Figure S2:** Pair distribution function g(r) with respect to distance (Å) for graphene sheet obtained. |

**Comparison with experiments**

Figures S3(a) and (b) show total correlation function (TCF) of simulated glassy graphene compared with experimetal TCF for irradiated graphene sheet obtained from the work of Eder *et al*^1^*.* We compare disordered graphene sheet obatined from cooling rate of 100 K/ps and 1000 K/ps with irradiated graphene sheet with 5.4% density deficit and 7.1% density deficit respectively. This is rationalized based on the fact that increased density deficit upon irradiation is similar to a lower density obtained upon faster cooling. We observe an excellent match with the experiments for GG obtained with cooling rates 100 K/ps and 1000 K/ps. Thus, we confirm that while cooling rate can have a notable effect on the structure of GG, the structures still exhibit a close match with the corresponding experimentally realized structures.

| 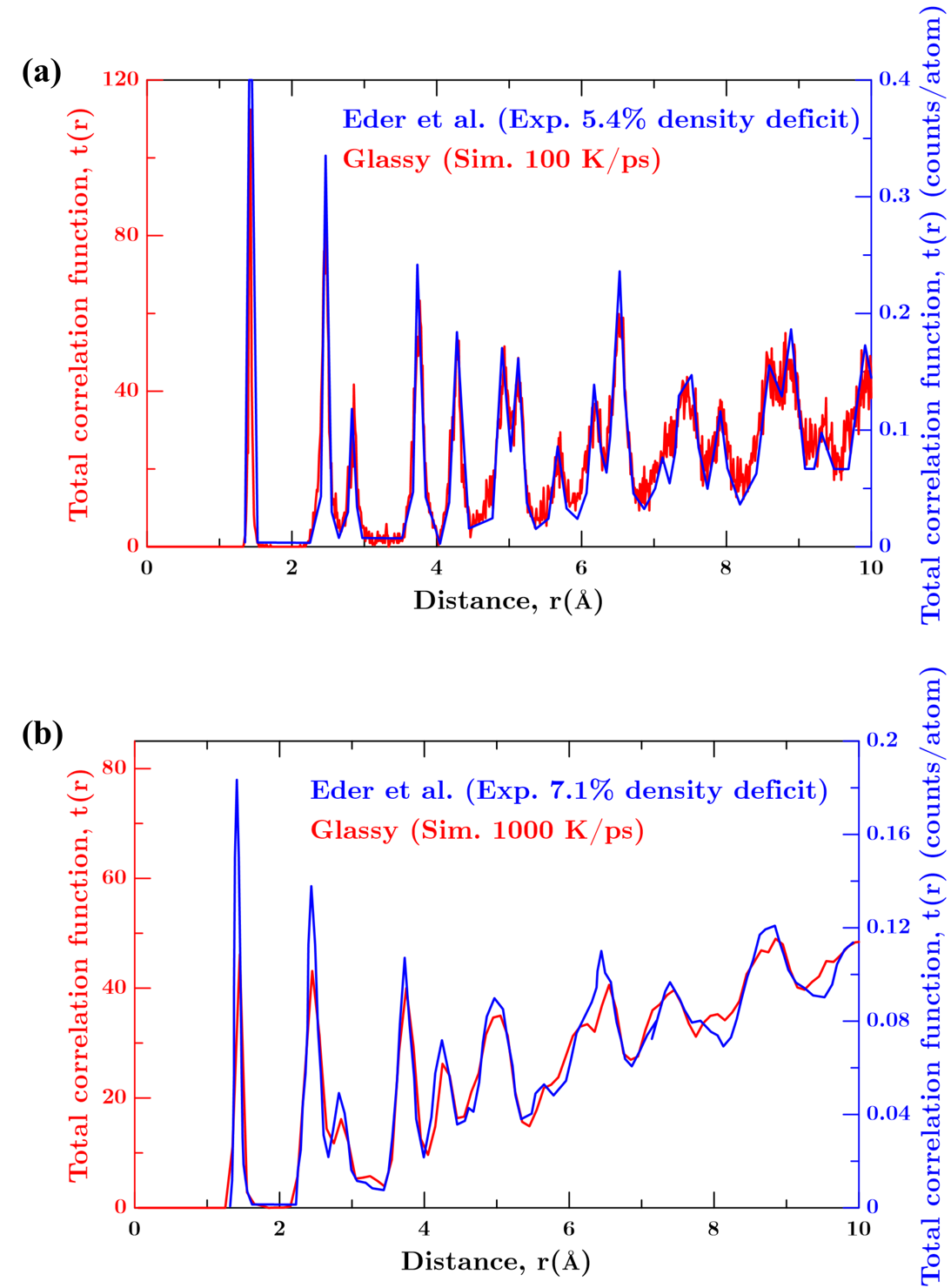 |
| --- |
|  |
| **Figure S3:** Total correlation function t(r) with respect to distance (Å) compared with irradiated graphene structure by Eder *et al*.^1^ |

**Reference**

1. Eder, F. R., Kotakoski, J., Kaiser, U. & Meyer, J. C. A journey from order to disorder — Atom by atom transformation from graphene to a 2D carbon glass. Scientific Reports **4**, 4060 (2014).
